# Effectiveness and Safety of Pharmacopuncture Therapy Compared to Standard Physical Therapy in Patients with Chronic Knee Pain: A Pilot Study for a Pragmatic Randomized Controlled Trial

**DOI:** 10.3390/medicina61061106

**Published:** 2025-06-18

**Authors:** Myung In Jeong, Jun Kyu Lim, Yong Jun Kim, Yu Sun Jeon, Suna Kim, Chang Youn Kim, Yeon-Cheol Park, Eun-Jung Kim, Yejin Hong, Dongwoo Nam, Yoon Jae Lee, Doori Kim, In-Hyuk Ha

**Affiliations:** 1 Department of Acupuncture & Moxibustion Medicine, Daejeon Jaseng Hospital of Korean Medicine, Daejeon 34127, Republic of Korea; aud07dls@gmail.com (M.I.J.); avici44@jaseng.org (C.Y.K.); 2 Department of Korean Internal Medicine, Daejeon Jaseng Hospital of Korean Medicine, Daejeon 34127, Republic of Korea; kcn1020@jaseng.org; 3 Department of Oriental Rehabilitation Medicine, Daejeon Jaseng Hospital of Korean Medicine, Daejeon 34127, Republic of Korea; azar20@jaseng.org; 4 Department of Gynecology of Korean Medicine, Daejeon Jaseng Hospital of Korean Medicine, Daejeon 34127, Republic of Korea; dbtjs1210@jaseng.org (Y.S.J.); tnsdk2648@jaseng.org (S.K.); 5 Department of Acupuncture & Moxibustion, College of Korean Medicine, Kyung Hee University, Kyung Hee University Hospital at Gangdong, Seoul 05278, Republic of Korea; icarus08@hanmail.net; 6 Department of Acupuncture & Moxibustion, Dongguk University Bundang Oriental Hospital, Seongnam-si 13601, Republic of Korea; hanijjung@naver.com; 7 Department of Acupuncture and Moxibustion, Kyung Hee University Korean Medicine Hospital, Seoul 02447, Republic of Korea; sky861@naver.com; 8 Department of Acupuncture & Moxibustion, College of Korean Medicine, Kyung Hee University, Seoul 02447, Republic of Korea; hanisanam@daum.net; 9 Jaseng Spine and Joint Research Institute, Jaseng Medical Foundation, Seoul 06110, Republic of Korea; goodsmile8119@gmail.com

**Keywords:** chronic pain, pragmatic RCT, PT, PPT

## Abstract

*Background and Objectives*: There have been a limited number of randomized controlled trials (RCTs) comparing pharmacopuncture therapy (PPT) and physical therapy (PT) for chronic knee pain. In this study, we assess the feasibility, safety, and preliminary effectiveness of PPT compared to PT in patients with chronic knee pain. *Materials and Methods*: This pilot study was designed as a two-arm, parallel RCT. Patients were recruited through in-hospital advertisements. Forty patients aged 19 to 70 with knee pain with a numeric rating scale (NRS) score of 5, persisting for >3 months, were randomized into the PPT or PT group. The type of PT solution or PT method was not determined in advance, leaving it to the clinician’s judgment. Treatment was administered twice weekly for 3 weeks with a 6-week follow-up. The primary outcome was the NRS score for knee pain, whereas the secondary outcomes were the visual analog scale (VAS), knee range of motion, Korean Western Ontario and McMaster (K-WOMAC), Patient Global Impression of Change, and five-level EuroQol five-dimension scores. Additionally, adherence, acceptability, dropout rate, and adverse events were measured to assess the feasibility of a follow-up main study. The protocol was registered at ClinicalTrials.gov (NCT06505681). *Results*: The PPT group showed significantly superior improvement compared with the PT group in the NRS (difference = −2.05, 95% confidence interval [CI]: −2.76 to −1.34), VAS (difference = −21.58, 95% CI: −29.42 to −13.74), and K-WOMAC scores (difference = −13.17, 95% CI: −21.67 to −4.67). Of the 55 patients who initially expressed interest in participation, 8 declined after receiving detailed information about this study. Among the forty enrolled participants, one patient in the PPT group dropped out, and one missed a single treatment session. Apart from these cases, all participants completed the assigned treatments and follow-up assessments, demonstrating high adherence. No serious adverse events were reported. *Conclusions*: PPT demonstrated excellent effectiveness in pain relief and functional improvement in these patients.

## 1. Introduction

Chronic knee pain is commonly caused by degenerative conditions such as osteoarthritis (OA), meniscal tears, and post-traumatic arthritis [1]. Patients with chronic knee pain often experience structural damage involving not only the articular cartilage but also the ligaments and bone–tendon junctions surrounding the knee joint [1]. The prevalence of OA, the most common cause of chronic pain, has been reported to be approximately 10% among Korean adults aged 20–89 years [1].

Treatment options for chronic knee pain vary depending on the underlying etiology. Conservative management—including exercise therapy, thermotherapy, electrotherapy, and pharmacological pain control—is generally considered the first line [2]. When these interventions fail to control symptoms or when joint deformities and functional limitations progress, surgical options such as osteotomy, arthroscopy, or total knee arthroplasty may be considered [3]. Surgical treatment options must be chosen carefully owing to the risks of complications, such as reoperation due to infection, pain, and functional impairment caused by patellar instability after total knee arthroplasty, as well as the limited lifespan of the replaced knee [4].

The standard treatment, or usual care, for patients with chronic knee pain typically combines various modalities, including pharmacological treatments and physical therapy. Studies have reported the effectiveness of transcutaneous electrical nerve stimulation (TENS), heat therapy, and interferential current therapy (ICT) in patients with chronic knee pain [5,6]. In Korea, superficial heat therapy, deep heat therapy, TENS, and ICT are frequently prescribed in clinical practice [7].

In addition to these standard treatments, traditional Korean medicine (KM) is a popular conservative treatment option for patients with knee pain, and studies have explored its effectiveness in managing knee pain [8]. Especially, pharmacopuncture therapy (PPT)—a technique involving the injection of herbal extracts into acupuncture points [9]—has gained widespread use for musculoskeletal disorders, including knee pain. PPT is thought to exert therapeutic effects through both pharmacological and neurophysiological mechanisms. Sweet Bee Venom is known for its anti-inflammatory and analgesic properties, partly mediated through the modulation of cytokines and neuropeptides [10,11]. Joongseongouhyul contains herbal components that promote blood circulation and reduce local stagnation, potentially alleviating chronic musculoskeletal pain [12].

Several studies have reported the clinical effectiveness of pharmacopuncture in reducing knee pain and improving functional outcomes [13,14,15]. However, most existing studies are limited by methodological weaknesses such as lack of randomization, small sample sizes, or reliance on subjective improvement rates without validated outcomes [16].

While KM-based interventions, including PPT, are widely accepted and reimbursed within the Korean healthcare system, these approaches remain underutilized and understudied in many other countries. As such, there is a clear need to build internationally relevant evidence to validate these treatments in broader clinical contexts.

Therefore, we conducted this pilot study to assess the feasibility, safety, and preliminary effectiveness of PPT compared to PT in patients with chronic knee pain. This study aims to inform the design of a larger-scale trial and contribute to bridging the global evidence gap for KM-based interventions.

## 2. Materials and Methods

### 2.1. Study Design

This two-arm, parallel, randomized, controlled pilot study enrolled 40 patients who were randomized at a 1:1 ratio into the PPT or control group at a Korean hospital. At the initial visit, the participants were provided with all necessary information about this study and completed an informed consent form. The assessors then screened the participants for eligibility based on the inclusion and exclusion criteria. Eligible participants were enrolled in this study. Treatment sessions were conducted twice weekly for 3 weeks, and the follow-up assessment was conducted 4 and 6 weeks after baseline. During the follow-up period, participants were observed through direct visits or telephone surveys.

All documents related to this study, including the study protocol, received approval from the local institutional review board prior to patient recruitment, and this study was conducted in accordance with the Declaration of Helsinki. Informed consent was obtained from all participants involved in this study. The protocol was registered at ClinicalTrials.gov (NCT06505681, registration date 17 July 2024).

### 2.2. Inclusion and Exclusion Criteria

The inclusion criteria were as follows: (1) numeric rating scale (NRS) score ≥ 5 for knee pain, (2) symptoms persisting for >3 months, (3) age between 19 and 70 years, and (4) patients who voluntarily decided to participate in this study and provided their written consent after fully understanding the study details.

The exclusion criteria were as follows: (1) patients diagnosed with a specific serious disease that may cause knee pain, including acute fracture, dislocation, and traumatic injury to ligaments and cartilage; (2) pain caused by conditions other than knee diseases, including tumor, fibromyalgia, rheumatoid arthritis, gout, and lumbar disc herniation; (3) patients whose knee pain started due to a traumatic event and required surgical intervention owing to suspected acute fracture or dislocation of the ligament or cartilage damage; (4) patients with other chronic diseases, including stroke, myocardial infarction, kidney disease, diabetic neuropathy, dementia, and epilepsy that may interfere with the interpretation of therapeutic effects or outcomes; (5) patients taking steroids, immunosuppressants, psychotropic medications, or other drugs that may affect the study outcomes; (6) patients for whom PPT use was inappropriate or unsafe, such as those with hemorrhagic disease, taking anticoagulant drugs, or with an increased risk of infection owing to severe diabetes mellitus; (7) patients who had taken medications that may have an impact on pain, such as nonsteroidal anti-inflammatory drugs, or received PPT or PT within 1 week; (8) pregnant women, those planning to get pregnant, or lactating women; (9) patients who had undergone knee surgery within 3 months or had knee replacement surgery performed; (10) patients who had participated in other clinical trials within 1 month, had participated in another study within 6 months from the date of screening, or planned to participate in other trials during the follow-up period of this trial; (11) participants who had difficulty completing the informed consent form; and (12) other patients whose participation in this study was deemed inappropriate by the investigator.

### 2.3. Interventions

#### 2.3.1. Control Group: Standard Physical Therapy (PT)

The control group visited the outpatient clinic twice weekly for 3 weeks post-enrollment. Although no predefined protocol was applied, most patients in the control group received transcutaneous electrical nerve stimulation (TENS) and superficial heat therapy, which are commonly used physical therapy modalities for knee pain in routine clinical practice in Korea. The selection of modalities, treatment area, and session duration were individualized based on the physician’s clinical judgment, considering the patient’s symptoms, radiographic findings, and degree of improvement.

#### 2.3.2. Experimental Group: PPT

The PPT group also visited the outpatient clinic twice weekly for 3 weeks post-enrollment and received PPT at each visit using a standardized disposable syringe (29-gauge × 13 mm insulin syringe). Insertion depth was approximately 1–1.5 cm, depending on the selected acupoint and the patient’s body type. The typical volume of pharmacopuncture solution administered per acupoint was approximately 1–2 mL; however, the final dosage was determined at the discretion of the physician based on the patient’s condition. Commonly used pharmacopuncture solutions for knee pain included Shinbaro (Jaseng Herbal Dispensary, Sungnam, Republic of Korea), Hwangryunhaedok, and Joongseongouhyul, all of which were selected according to the physician’s clinical diagnosis and pattern identification.

All procedures were performed by licensed Korean medicine doctors (KMDs) with over 3 years of clinical experience. Participants were monitored for adverse events throughout the intervention period. The acupoints used for PPT, along with the type and total dose of the pharmacopuncture solution, were documented.

### 2.4. Outcomes

Follow-up assessments were conducted up to week 6. The primary endpoint was defined as week 4, immediately after the completion of all treatment sessions. NRS, VAS, and ROM were assessed weekly during the treatment period (baseline, week 2, and week 3) and twice during the follow-up period (weeks 4 and 6). K-WOMAC, SF-12, and EQ-5D-5L were measured at baseline and at two follow-up time points (weeks 4 and 6). The surveys were administered by a KMD trained in the analysis methods to achieve consistency in the measurement results. To ensure objective assessment, the assessor was not involved in the interventions but performed the assessment and recorded the results.

#### 2.4.1. Primary Outcome: NRS Score

The severity of knee pain was assessed using the NRS, which was developed to represent subjective pain experienced by patients. The participants were instructed to rate the intensity of their knee pain at each visit by selecting a number on a scale ranging from 0 (no pain or discomfort) to 10 (worst pain or discomfort imaginable) [17].

#### 2.4.2. Secondary Outcomes

##### Visual Analog Scale (VAS) Score

The VAS, a scale developed to represent subjective pain experienced by the patient, was employed to assess knee pain severity. The participants were instructed to indicate the intensity of their knee pain at the first visit of each week by marking on a 100 mm horizontal line with one end indicating “no pain” and the other indicating “worst pain imaginable” [18].

##### Range of Motion (ROM) of the Knee

To assess knee joint movement before and after treatment, active ROM was measured at the initial visit each week during the intervention period and at weeks 4 and 6. Active ROM was assessed by measuring the angle between the participant’s leg and an imaginary line drawn perpendicular to the ground during the maximum ROM of the participant’s knee in flexion, extension, left lateral flexion, and right lateral flexion. If the measurement was not possible because of pain, the ROM was recorded as uncheckable.

##### Korean Western Ontario and McMaster (K-WOMAC) Score

The K-WOMAC score was used as a functional outcome measure for the participants at baseline and weeks 4 and 6, which were the post-intervention time points. This 24-item questionnaire was developed to assess the degree of functional impairment caused by knee pain, with higher scores indicating more severe functional impairment. Each question is rated on a five-level scale, and the instrument consists of three subscales: pain (5 items), stiffness (2 items), and functional limitations (17 items) [19].

##### Patient Global Impression of Change Score

The Patient Global Impression of Change was used for the subjective assessment of the participants’ impressions of improvement in their symptoms at seven levels: 1, very much improved; 2, much improved; 3, minimally improved; 4, no change; 5, minimally worse; 6, much worse; and 7, very much worse [20].

##### Short Form-12 Health Survey (SF-12) Version 2 Score

To monitor changes in the health-related quality of life (HRQoL) of the participants, the SF-12 version 2 was used at baseline and at weeks 4 and 6, which were the post-intervention time points. The questionnaire comprises 12 items in 8 categories: physical functioning, physical role, bodily pain, general health, vitality, social functioning, emotional role, and mental health, with higher scores indicating better HRQoL. The Korean version of the SF-12, whose reliability and validity for the assessment of HRQoL have been verified in a previous study [21], was used in this study.

##### Five-Level EuroQol 5-Dimension (EQ-5D-5L) Score

The EQ-5D-5L is an instrument developed by the EuroQol group commonly employed in the healthcare sector owing to its proven objectivity and validity for assessing HRQoL. The EQ-5D-5L consists of questions across five domains: mobility, self-care, usual activities, pain/discomfort, and anxiety/depression. Each item is rated as one of five levels: 1, no problems; 2, slight problems; 3, moderate problems; 4, severe problems; and 5, extreme problems. This questionnaire was administered at the first visit and at weeks 4 and 6 [22].

##### Feasibility-Related Outcomes

To assess the feasibility of the trial design, the following indicators were evaluated: (1) adherence, defined as the proportion of participants who completed all scheduled intervention sessions and follow-up visits; (2) acceptability, assessed indirectly through the number of missed visits not due to clinical deterioration; and (3) dropout rate, defined as the proportion of participants who withdrew from this study after randomization. These parameters were monitored throughout the study period and used to evaluate the practical applicability and tolerability of the intervention in a real-world clinical setting.

##### Drug Consumption

The types and doses of medications taken by the participants during the study period were recorded during the visits. Regarding the types of medications, all medications prescribed for the target disease or taken for symptoms related to this study were identified.

##### Adverse Events

Liver function tests (total protein, albumin, total bilirubin, alkaline phosphatase, alanine transaminase, aspartate transaminase, and gamma-glutamyl transpeptidase) and kidney function tests (blood urea nitrogen and creatinine) were conducted in both groups before and after treatment for safety assessments. Blood samples obtained by venipuncture were discarded immediately after analysis in accordance with the standard operating procedure for disposal by the laboratory testing team.

AEs were defined as unintended signs, symptoms, or diseases occurring after treatment, including those not necessarily related to the intervention. AEs were collected on the basis of the patient-reported symptoms and investigator observation. AEs suspected to be related to treatment, abnormal test results, and the incidence of serious AEs were analyzed.

Causality between the treatment and AEs was assessed using the six-level scale of the World Health Organization–Uppsala Monitoring Center Causality Assessment System (1, definitely related; 2, probably related; 3, possibly related; 4, probably unrelated; 5, definitely unrelated; and 6, unknown).

The severity of AEs was assessed at three levels based on the Spilker classification: (1) mild (no treatment is required, and the AE does not substantially interfere with activities of daily living [ADLs]), (2) moderate (the AE considerably interferes with ADLs, may require treatment, and may resolve after treatment), and (3) severe (the AE requires immediate advanced treatment and may have sequelae due to the severity of symptoms).

### 2.5. Sample Size Calculation

No previous study has compared the effectiveness of PPT and PT in patients with chronic knee pain to serve as the basis for sample size calculation. As this was a pilot study aimed at assessing the feasibility of a future main study, the minimum number considered acceptable for a pilot study—15 participants per group—was used [23,24]. Assuming a 25% dropout rate and considering the need for subgroup analysis, we recruited 20 patients for each group (total of 40 patients).

### 2.6. Recruitment

Participants were recruited through in-hospital advertisements, including posters and bulletin board notices. Participation was entirely voluntary, and no direct invitations or referrals were issued by clinicians. Some patients enrolled after noticing the advertisement during routine clinical visits, while others participated upon hearing about this study from acquaintances.

### 2.7. Randomization

Eligible participants were randomly assigned to the PPT (n = 20) or control group (n = 20) using a random number table generated with RStudio (version 1.1 463; RStudio, Inc., Boston, MA, USA). Random sequence generation was based on block randomization, with block sizes of two or four randomly selected. The randomization results were placed in a sealed opaque envelope and stored in a dual-lock cabinet managed by a third party not involved in this study. The investigator opened the sealed randomization envelopes only after confirming eligibility for this study and obtaining informed consent. Group assignments and randomization numbers were recorded in the electronic medical record system.

### 2.8. Blinding

Owing to the study design and nature of the intervention, blinding of the participants and KMDs was not possible. Nevertheless, to minimize the risk of bias, outcome assessors were blinded and conducted assessments independently.

### 2.9. Data Collection and Management

An Excel case report form (CRF) (Microsoft, Redmond, WA, USA) was used in this study, and data validation was performed through query checks. The Excel CRF was locked after data cleaning, and no research investigators, except for data manager, had access to the data.

### 2.10. Statistical Analysis

Intention-to-treat (ITT) analysis was used as the primary analysis, and per-protocol (PP) analysis was performed as a sensitivity analysis for patients who completed five or more treatment sessions during a 3-week treatment period.

For the linear mixed model (LMM), which was the main analysis, a repeated-measures approach was used to handle missing data. For analysis using analysis of covariance (ANCOVA) and comparison of the area under the curve (AUC), which were secondary analyses, multiple imputation (MI) was applied to address missing data. An additional sensitivity analysis was used in the last observation carried forward (LOCF) imputation.

Participants’ baseline characteristics were compared between the two groups. Continuous variables are expressed as mean (standard deviation) or median (quartile), and the differences between the two groups were compared using the Student *t* test or Wilcoxon rank-sum test, depending on the data distribution. Categorical variables are expressed as frequency (%), and the two groups were compared using the chi-square or Fisher exact test.

Differences in changes from baseline at each time point in the primary (NRS score) and secondary outcomes (VAS, ROM, K-WOMAC, EQ-5D-5L, and SF-12 values) were analyzed using LMM. Baseline values for each outcome variable were included as covariates in both LMM and ANCOVA models. Other baseline characteristics (age, sex, BMI) were not included as covariates, as there were no significant differences between groups. ANCOVA was performed on the datasets processed using MI and LOCF.

To compare the cumulative effects during the treatment and total study periods, AUC values at each time point after randomization were calculated, and the difference between the groups was analyzed using the Student *t* test.

“Recovery” was defined as ≥50% reduction in the NRS score of knee pain. The Kaplan–Meier survival curve and log-rank test were used to compare the time to recovery between the groups. Hazard ratios were estimated using the Cox proportional hazards model.

All effect estimates were presented with 95% confidence intervals to emphasize estimation over hypothesis testing, which was in line with the pilot nature of the study. The significance level was set at 0.05, and all statistical analyses were performed using SAS (version 9.4; SAS Institute, Inc., Cary, NC, USA) and RStudio (version 1.1.463; RStudio, Inc.).

## 3. Results

### 3.1. Participant Flow

**Figure 1:**
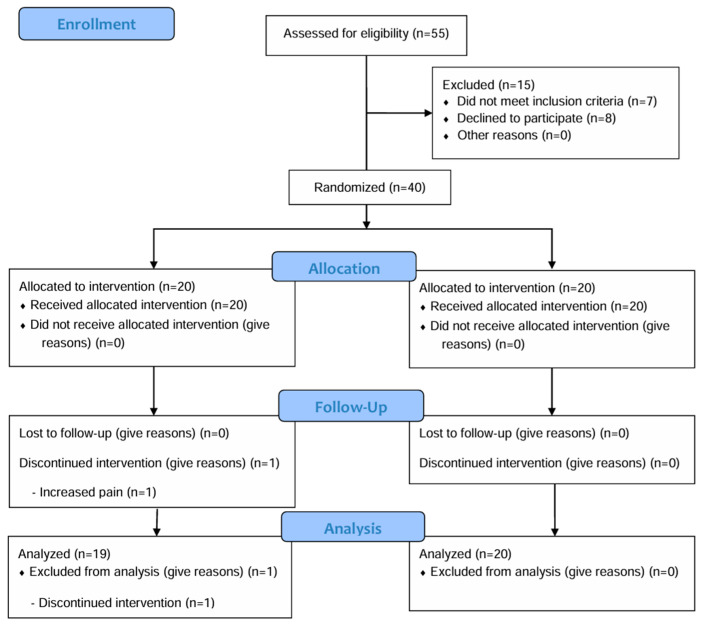
Participant flow chart.

From March 2023 to June 2023, 55 patients underwent screening (Figure 1), of which 15 were excluded based on the exclusion criteria. Finally, 40 patients were enrolled in this study and randomly assigned to either the PPT (n = 20) or standard PT group (control, n = 20). Following randomization, one patient in the PPT group withdrew from this study due to increased pain. Consequently, ITT analysis was performed on 39 participants.

### 3.2. Baseline Characteristics

Of the 39 participants, 17 (43.6%) were male and 22 (56.4%) were female. The mean age was approximately 44 years, and the mean body mass index (BMI) was 22.24 ± 3.69 kg/m^2^. In the analysis of the baseline characteristics of the PPT (n = 19) and control (n = 20) groups, no statistically significant differences were observed in age, weight, BMI, body temperature, NRS score, VAS score, ROM, K-WOMAC score, and EQ-5D-5L score between the two groups. During the screening process, three participants in the PPT group and two in the control group showed abnormal findings on knee radiographs; however, these results were not considered clinically significant (Table 1).

### 3.3. Treatment

The most commonly used pharmacopuncture solution in the PPT group was Shinbaro, which was administered to 17 participants (89.5%) an average of 5.41 ± 1.54 times. Other types of pharmacopuncture solutions administered in the PPT group included Harpagoside (eight participants, 42.1%), Placenta Hominis (four participants, 21.1%), Joongseongouhyul (five participants, 26.3%), and Huang-Lian-Jie-Du-Tang (six participants, 31.6%). All participants in the PT group underwent TENS and heat therapy (Table S1).

### 3.4. Primary and Secondary Outcomes

**Figure 2:**
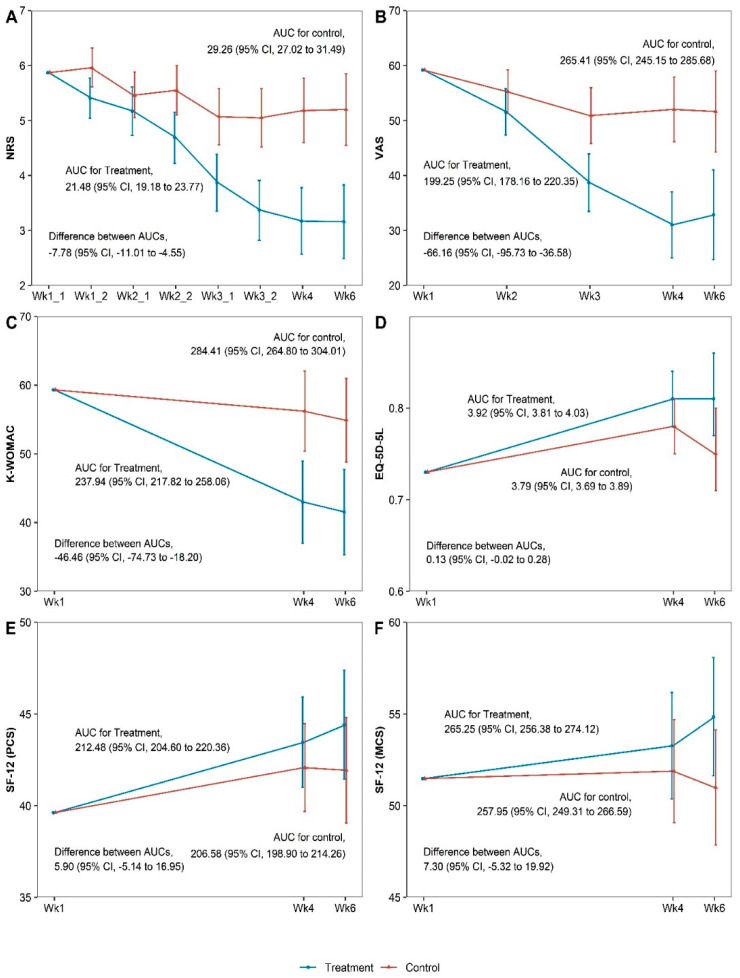
Areas under the curve for the outcomes at week 6 for (**A**) Numeric rating scale, (**B**) Visual analogue scale, (**C**) Korean Western Ontario and McMaster, (**D**) Five-level EuroQol 5 dimension, (**E**) Physical component summery, (**F**) Mental component summary. AUC, area under the curve; CI, confidence interval; EQ-5D-5L, five-level EuroQol 5 dimensions; K-WOMAC, Korean Western Ontario and McMaster; MCS, mental component summary; NRS, numeric rating scale; PCS, physical component summary; SF-12, Short Form-12 Health Survey version 2; VAS, visual analog scale.

The PPT group showed greater improvement in all outcomes at the primary endpoint (week 4) and at week 6 follow-up than the control group (Table 2). In particular, at the primary endpoint, the PPT group showed significant improvements in the NRS (difference = −2.05, 95% confidence interval [CI]: −2.76 to −1.34, *p* < 0.001), VAS (difference = −21.58, 95% CI: −29.42 to −13.74, *p* < 0.001), and K-WOMAC scores (difference = −13.17, 95% Cl: −21.67 to −4.67, *p* = 0.003) compared with the control group. These significant differences in effectiveness were maintained until week 6. Outcome measurements at all time points are presented in Table S2. Similar patterns were confirmed by the results of sensitivity ANCOVA analyses (Tables S3 and S4). Regarding the cumulative effect at week 6 based on the AUC calculation, the PPT group showed statistically significant effectiveness compared with the control group in terms of outcome measures such as the NRS, VAS, and K-WOMAC scores (Figure 2). The ITT analysis results are outlined in Table 2.

### 3.5. Survival Analysis

**Figure 3:**
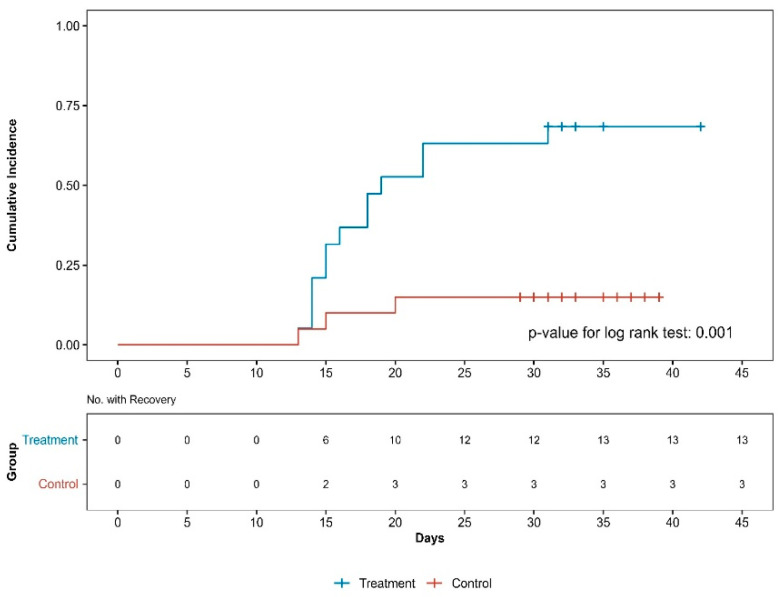
Cumulative incidence curves of recovery by group. No., number.

Survival analysis confirmed that the PPT group had a shorter recovery time than the control group (*p* < 0.001, log-rank test; Figure 3). The hazard ratio of the PT group to the PPT group using the Cox regression model was 0.16 (95% CI: 0.04–0.65, *p* < 0.015, *p* < 0.001 by log-rank test). The number of patients who recovered by the last follow-up was 13 in the PPT group and 3 in the PT group.

### 3.6. Feasibility-Related Outcomes

Of the 55 patients who initially expressed interest in participation, 8 declined after receiving detailed information about the study. Of the 40 randomized participants, 38 (95.0%) completed all six scheduled treatment sessions and follow-up assessments. One participant (2.5%) in the PPT group discontinued treatment after the first session due to increased pain, and one additional patient missed a single session but completed all other visits. The mean number of sessions in the PPT group was 5.7 out of 6 (SD = 1.13), indicating a high adherence rate. The mean number of sessions in the PT group was 6.0. The overall dropout rate was 2.5%, and no participants withdrew due to dissatisfaction or adverse events. These findings demonstrate the excellent feasibility and acceptability of both interventions in a clinical setting.

### 3.7. Adverse Events

AEs were reported in eight and five patients in the PPT and control groups, respectively. AEs with causality to the study intervention were identified only in the PPT group; the types of AEs included paresthesia and bruising. The severity of AEs was moderate or mild (Table 3).

Regarding laboratory test results, there were no significant changes in the test results for each test item before and after treatment, and there were no significant differences in the ratio of abnormalities in the test results before and after treatment (Tables S5 and S6).

## 4. Discussion

This pilot study evaluated the feasibility, safety, and preliminary effectiveness of PPT compared to PT in patients with chronic knee pain. The findings suggest favorable trends in pain reduction and functional improvement associated with PPT, supporting the need for investigation in a larger, adequately powered trial.

These significant differences have meaningful implications for the patient-acceptable symptom state (PASS) and minimal clinically important difference (MCID). PASS refers to the state of symptoms a patient considers acceptable and indicates the subjective intensity of symptoms that the patient deems satisfactory. Tubach et al. reported that the PASS of patients with knee OA was reached when the VAS score was reduced to 32.3 [25]. The VAS score at week 4 in the PPT group of the present study was 30.63, which is lower than the suggested PASS. Furthermore, the MCID, which is the minimal change that a patient perceives as meaningful improvement, serves as a key indicator for evaluating the clinical importance of improvement in function and pain [26]. Kim et al. reported that the MCID of WOMAC scores before and after medial opening wedge high tibial osteotomy was 16.1 points [26]. In the present study, the change in K-WOMAC score in the PPT group at week 4 was approximately 16.29, exceeding the reported MCID value. Therefore, the patients in the PPT group achieved an improvement in clinical importance following treatment. In contrast, the post-treatment change in the PT group was not significant, and the difference did not meet the conditions presented in previous studies.

The PPT employed in this study was a KM treatment modality that combines the physical stimulation of traditional acupuncture and the chemical and pharmacological actions of herbal medicinal extracts [9,27,28]. PPT contributes to improving pain by alleviating nerve entrapment, restoring the disturbed homeostasis of the tissues and nerves around the knee, and reducing inflammation [29,30,31,32]. In this study, different types of pharmacopuncture solutions, including Shinbaro, Harpagoside, Huang-Lian-Jie-Du-Tang, Joongseongouhyul, and Placenta Hominis, were selectively used according to the clinical judgment of KMDs, with Shinbaro being the most frequently used.

In a previous study, Shinbaro was shown to reduce inflammation through multiple mechanisms: suppressing proteoglycan degradation; downregulating inflammatory mediators such as inducible nitric oxide synthase (iNOS), cyclooxygenase-2 (COX-2), and tumor necrosis factor-alpha (TNF-α); increasing the peripheral pain threshold; activating alkaline phosphatase in osteoblasts; and inhibiting the activity of matrix metalloproteinases (MMP-2 and MMP-9) [30,31]. The mechanism of Shinbaro indicates that the active pharmacological ingredient of the pharmacopuncture solution may have contributed to the excellent outcomes observed in the PPT group.

Furthermore, chronic knee pain is increasingly understood within a multifactorial framework, involving not only joint-level degeneration but also systemic low-grade inflammation, gut–joint axis interactions, and environmental influences. Recent studies suggest that gut microbiota-derived tryptophan metabolites may influence osteoarthritis-related pain and inflammatory profiles [33], while long-term environmental factors such as climate and ambient conditions have also been shown to shape chronic musculoskeletal pain trajectories [34]. These findings support the rationale for integrative therapies such as PPT, which may exert effects through both local and systemic pathways.

Another important point is that the study participants experienced chronic knee pain (NRS score ≥ 5 for >3 months), and approximately 87% had normal findings on knee radiographs. The progression of knee osteoarthritis (OA) is typically classified using the Kellgren–Lawrence grading system, and treatment decisions vary based on functional status, disease severity, and underlying pathology [35]. However, in the early stages of knee OA, radiographic changes may be minimal or absent, even when patients experience intermittent knee pain, especially with physical activity [36].

Thus, normal radiographic findings should not delay treatment. Early intervention during the so-called “window of opportunity,” as described by Mahmoudian et al. [36], may help prevent progression to more severe OA. This window represents a critical period in early symptomatic knee OA where timely intervention can halt or slow disease progression. Despite its importance, few studies have targeted patients in this early phase without radiographic progression. Most prior research has focused on individuals with established radiographic OA or advanced disease [37,38]. Therefore, this study is meaningful in that it includes patients within this under-researched early stage, offering insight into treatment strategies that may prevent long-term deterioration.

In analyzing the AEs reported in this study, 3 of 19 participants in the PPT group complained of bruising at the site of PPT administration, which was determined to be causally related to the treatment. The size and duration of the bruises varied among individuals; however, the severity of the AEs was considered mild, and the AEs disappeared within 2 weeks. No systemic adverse drug reactions or clinically significant changes in laboratory test results were observed in the PPT group. In contrast, several AEs have been reported with intra-articular corticosteroid injections, such as bruising, swelling, rapid destruction of articular cartridges with repeated injections, and even osteonecrosis [39]. These findings support the potential of PPT as a generally well-tolerated treatment. Nonetheless, larger studies with longer follow-ups are necessary to confirm its safety profile.

Based on the findings of this pilot trial, a sample size calculation was performed to inform the design of a future main study. Using the NRS score at week 4 as the primary endpoint, the between-group difference in means was −2.05, with a pooled standard deviation of approximately 1.13, yielding an estimated effect size of 1.81, which is considered large. Assuming a two-sided significance level of 0.05 and a power of 90%, the calculated sample size required per group is 8 participants, for a total of 16. This number is smaller than the actual number recruited in this pilot study (N = 40), indicating that the trial was adequately powered for feasibility assessment. Nevertheless, these parameters provide a rational basis for planning a main RCT, although more conservative effect size estimates and longer-term follow-up would be advisable to ensure generalizability.

This study has several limitations inherent to its pilot design. First, the small sample size and wide confidence intervals limit the generalizability and precision of the findings. Second, long-term outcomes beyond the 6-week follow-up period were not assessed. Third, while adherence was monitored through attendance, no formal patient-reported acceptability or adherence measures were collected. These limitations should be addressed in future main trials. Lastly, although PPT was delivered based on clinical routine, no formal standard operating procedure or protocolized pattern identification was used. Treatment decisions were made by experienced KMDs according to shared clinical practices, which may limit replicability in other settings. However, although treatment was individualized, acupoints commonly indicated for knee pain (such as ST35, EX-LE4, and GB34) and frequently used pharmacopuncture solutions (e.g., Shinbaro, Hwangryunhaedok) were consistently selected across patients. This approach reflects real-world practice while maintaining a reasonable degree of protocol consistency.

This study suggested that PPT can serve as an effective and safe treatment option for patients with chronic knee pain, providing essential evidence to guide the decision to proceed with a larger main study. Additionally, as radiographic progression was not included in the eligibility criteria of the participants, the results are applicable to various types of patients with knee pain commonly encountered in the primary care setting.

## 5. Conclusions

This pilot study found that PPT led to greater improvements in pain and functional outcomes compared to standard PT among patients with chronic knee pain. While the results should be interpreted with caution due to the limited sample size, they offer meaningful insights to support the feasibility of a future full-scale trial. These findings may help inform clinical decision-making and future research on integrative approaches to managing chronic knee pain.

## Figures and Tables

**Table 1:** Baseline characteristics.

	Total	PT Group	PPT Group	*p*-Value
	N = 39	n = 19	n = 20	
Sex				
Male	17 (43.6)	9 (47.4)	8 (40.0)	0.888
Female	22 (56.4)	10 (52.6)	12 (60.0)	
Age, years				
Mean ± SD	43.92 ± 15.26	45.05 ± 16.34	42.85 ± 14.50	0.658
<30	10 (25.6)	6 (31.6)	4 (20.0)	0.288
30–39	9 (23.1)	3 (15.8)	6 (30.0)	
40–49	3 (7.7)	-	3 (15.0)	
50–59	8 (20.5)	4 (21.1)	4 (20.0)	
≥60	9 (23.1)	6 (31.6)	3 (15.0)	
BMI, kg/m^2^				
Mean ± SD	22.24 ± 3.69	22.47 ± 3.68	22.02 ± 3.78	0.705
<25	30 (76.9)	13 (68.4)	17 (85.0)	0.273
≥25	9 (23.1)	6 (31.6)	3 (15.0)	
Knee radiograph findings				
normal	34 (87.2)	16 (84.2)	18 (90.0)	0.661
NCS	5 (12.8)	3 (15.8)	2 (10.0)	
Pain location				
Lt. knee	18 (46.2)	9 (47.4)	9 (45.0)	1.000
Rt. knee	21 (53.8)	10 (52.6)	11 (55.0)	
Symptom severity				
Mild (not uncomfortable)	-	-	-	0.431
Moderate (uncomfortable but manageable)	22 (56.4)	9 (47.4)	13 (65.0)	
Severe (serious but untreated)	17 (43.6)	10 (52.6)	7 (35.0)	
Severe (serious and requires treatment)	-	-	-	
Medication history				
Yes	22 (56.4)	11 (57.9)	11 (55.0)	1.000
No	17 (43.6)	8 (42.1)	9 (45.0)	

Abbreviations: BMI, body mass index; Lt., left; PPT, pharmacopuncture; PT, physical therapy; Rt., right; SD, standard deviation.

**Table 2:** Primary and secondary outcomes after treatment at each time point.

		Week 1-1	Week 2-1	Week 3-1	Week 4	Week 6
NRS score	PPT group	5.87 (5.62–6.12)	5.16 (4.64–5.67)	3.89 (3.38–4.39)	3.15 (2.64–3.66)	3.15 (2.64–3.66)
	PT group		5.45 (4.96–5.95)	5.05 (4.56–5.55)	5.20 (4.71–5.70)	5.20 (4.71–5.70)
	Difference	—	−0.29 (−1.01 to 0.42)	−1.17 (−1.88 to −0.46)	−2.05 (−2.76 to −1.34)	−2.05 (−2.76 to −1.34)
	*p*-value	—	0.417	0.002 **	<0.001 ***	<0.001 ***
VAS score	PPT group	59.18 (56.77–61.59)	51.40 (45.72–57.09)	38.74 (33.16–44.32)	30.63 (25.05–36.21)	32.17 (25.98 to 38.35)
	PT group		54.97 (49.51–60.42)	50.67 (45.21–56.12)	52.22 (46.76–57.67)	50.92 (44.93 to 56.91)
	Difference	—	−3.56 (−11.48 to 4.35)	−11.93 (−19.77 to −4.09)	−21.58 (−29.42 to −13.74)	−18.75 (−27.39 to −10.11)
	*p*-value	—	0.373	0.003 **	<0.001 ***	<0.001 ***
K-WOMAC score	PPT group	59.31 (53.69–64.92)	—	—	43.02 (36.96–49.07)	41.49 (35.43–47.55)
	PT group		—	—	56.18 (50.28–62.09)	54.93 (49.03–60.84)
	Difference	—	—	—	−13.17 (−21.67 to −4.67)	−13.45 (−21.95 to −4.94)
	*p*-value	—	—	—	0.003 **	0.003 **
PCS score	PPT group	39.62 (37.67–41.57)	—	—	43.52 (40.84–46.20)	44.35 (41.67–47.03)
	PT group		—	—	42.03 (39.41–44.64)	42.00 (39.39–44.62)
	Difference	—	—	—	1.49 (−2.26 to 5.25)	2.35 (−1.41 to 6.10)
	*p*-value	—	—	—	0.429	0.216
MCS score	PPT group	51.48 (48.60–54.36)	—	—	53.27 (50.28–56.26)	54.85 (51.86–57.84)
	PT group		—	—	51.89 (48.97–54.80)	51.01 (48.09–53.92)
	Difference	—	—	—	1.39 (−2.84 to 5.61)	3.85 (−0.38 to 8.07)
	*p*-value	—	—	—	0.515	0.074
EQ-5D-5L score	PPT group	0.73 (0.69–0.76)	—	—	0.81 (0.77–0.85)	0.81 (0.77–0.85)
	PT group		—	—	0.78 (0.74–0.82)	0.75 (0.72–0.79)
	Difference	—	—	—	0.03 (−0.02 to 0.08)	0.06 (0.01–0.11)
	*p*-value	—	—	—	0.286	0.030 *

The differences between the two groups in the changes from baseline at each time point were analyzed using LMM, wherein the baseline value of each outcome was used as a covariate, and the group was used as a fixed factor. Week 1-1 is the time point for baseline measurements, and a week-4 follow-up after 3 weeks of intervention is the time point for assessing the primary endpoint. * *p* < 0.05, ** *p* < 0.01, *** *p* < 0.001. Abbreviations: EQ-5D-5L, five-level EuroQol 5 dimensions; K-WOMAC, Korean Western Ontario and McMaster; MCS, mental component summary; NRS, numeric rating score; PCS, physical component summary; PPT, pharmacopuncture therapy; PT, physical therapy; VAS, visual analog scale.

**Table 3:** Adverse events.

Group	Adverse Event (LLT)	Severity	Causality
PT	Bruise	Moderate	Certain
PT	Bruise	Moderate	Certain
PT	Paresthesia	Mild	Possible
PT	Bruise	Mild	Certain

PT, pharmacopuncture; LLT, lowest-level term.

## Data Availability

The data that support the findings of this study are available on request from the corresponding author. The data are not publicly available owing to privacy/ethical restrictions.

## Supplementary Materials

The following supporting information can be downloaded at https://www.mdpi.com/article/10.3390/medicina61061106/s1. Table S1: Treatment details; Table S2: Primary and secondary outcomes after treatment at each time point (linear mixed model); Table S3: Primary and secondary outcomes after treatment at each time point (MI ANCOVA); Table S4: Primary and secondary outcomes after treatment at each time point (LOCF ANCOVA); Table S5: Laboratory test results before and after the intervention; Table S6: Ratios of abnormality in the laboratory test results.

## Abbreviations

The following abbreviations are used in this manuscript:

OA	osteoarthritis
TENS	transcutaneous electrical nerve stimulation
ICT	interferential current therapy
KM	Korean medicine
PPT	pharmacopuncture therapy
RCTs	randomized controlled trials
NRS	numeric rating scale
PT	physical therapy
KMDs	KM doctors
VAS	visual analog scale
ROM	range of motion
K-WOMAC	Korean Western Ontario and McMaster
SF-12	Short Form-12 Health Survey
HRQoL	health-related quality of life
EQ-5D-5L	five-level EuroQol 5 dimensions
AEs	adverse events
ADLs	activities of daily living
CRF	case report form
ITT	Intention to treat
PP	per protocol
LMM	linear mixed model
ANCOVA	analysis of covariance
AUC	area under the curve
LOCF	last observation carried forward
CI	confidence interval
PASS	patient-acceptable symptom state
MCID	minimal clinically important difference
MMP	matrix metalloproteinase
