# Supplementary material for: Effectiveness and Safety of Pharmacopuncture Therapy Compared to Standard Physical Therapy in Patients with Chronic Knee Pain: A Pilot Study for a Pragmatic Randomized Controlled Trial
Source: Medicina (Kaunas). 2025 Jun 18;61(6):1106. doi: 10.3390/medicina61061106 (PMC12195255; doi:10.3390/medicina61061106)
Supplement: Supplementary file 1 [file medicina-61-01106-s001.zip › medicina-3684520-supplementary.pdf]

Supplementary Materials

Effectiveness and Safety of Pharmacopuncture Therapy Compared to Standard Physical Therapy in Patients with Chronic Knee Pain: A Pilot Study for a Pragmatic Randomised Controlled Trial

Table S1. Treatment details

	N (%)	Number of treatments (mean ± SD)
PPT group		
Shinbaro	17 (89.5)	5.41 ± 1.54
Harpagoside	8 (42.1)	3.13 ± 1.96
Huang-Lian-Jie-Du-Tang	6 (31.6)	1.17 ± 0.41
Jungsongouhyul	5 (26.3)	1.00 ± 0.00
Placenta Hominis	4 (21.1)	3.75 ± 2.22
PT group		
TENS	20 (100)	6.00 ± 0.00
HP	20 (100)	6.00 ± 0.00

Abbreviations: HP, hot pack; PPT, pharmacopuncture; PT, physical therapy; SD, standard deviation; TENS, transcutaneous electrical nerve stimulation

Table S2. Primary and secondary outcomes after treatment at each timepoint (linear mixed model)

		Week 1-1	Week 1-2	Week 2-1	Week 2-2	Week 3-1	Week 3-2	Week 4	Week 6
NRS score	PPT group	5.87 (5.62–6.12)	5.41 (4.91–5.92)	5.16 (4.64–5.67)	4.68 (4.17–5.18)	3.89 (3.38–4.39)	3.36 (2.85–3.87)	3.15 (2.64–3.66)	3.15 (2.64–3.66)
	PT group		5.95 (5.46–6.45)	5.45 (4.96–5.95)	5.55 (5.06–6.05)	5.05 (4.56–5.55)	5.05 (4.56–5.55)	5.20 (4.71–5.70)	5.20 (4.71–5.70)
	Difference	—	–0.54 (–1.25 to 0.17)	–0.29 (–1.01 to 0.42)	–0.88 (–1.59 to –0.17)	–1.17 (–1.88 to –0.46)	–1.69 (–2.40 to –0.98)	–2.05 (–2.76 to –1.34)	–2.05 (–2.76 to –1.34)
	p-value	—	0.135	0.417	0.016*	0.002**	< 0.001***	< 0.001***	< 0.001***
VAS score	PPT group	59.18 (56.77–61.59)		51.40 (45.72–57.09)		38.74 (33.16–44.32)		30.63 (25.05–36.21)	32.17 (25.98–38.35)
	PT group			54.97 (49.51–60.42)		50.67 (45.21–56.12)		52.22 (46.76–57.67)	50.92 (44.93–56.91)
	Difference	—		–3.56 (–11.48 to 4.35)		–11.93 (–19.77 to –4.09)		–21.58 (–29.42 to –13.74)	–18.75 (–27.39 to –10.11)
	p-value	—		0.373		0.003**		< 0.001***	< 0.001***
K-WOMAC score	PPT group	59.31 (53.69–64.92)						43.02 (36.96–49.07)	41.49 (35.43–47.55)
	PT group							56.18 (50.28–62.09)	54.93 (49.03–60.84)

	Difference	—		–13.17 (–21.67 to –4.67)	–13.45 (–21.95 to –4.94)
	p-value	—		0.003**	0.003**
PCS score	PPT group	39.62 (37.67–41.57)		43.52 (40.84–46.20)	44.35 (41.67–47.03)
	PT group			42.03 (39.41–44.64)	42.00 (39.39–44.62)
	Difference	—		1.49 (–2.26 to 5.25)	2.35 (–1.41 to 6.10)
	p-value	—		0.429	0.216
MCS score	PPT group	51.48 (48.60–54.36)		53.27 (50.28–56.26)	54.85 (51.86–57.84)
	PT group			51.89 (48.97–54.80)	51.01 (48.09–53.92)
	Difference	—		1.39 (–2.84 to 5.61)	3.85 (–0.38 to 8.07)
	p-value	—		0.515	0.074
EQ-5D-5L score	PPT group	0.73 (0.69–0.76)		0.81 (0.77–0.85)	0.81 (0.77–0.85)
	PT group			0.78 (0.74–0.82)	0.75 (0.72–0.79)

Difference	—		0.03 (–0.02 to 0.08)	0.06 (0.01–0.11)
p-value	—		0.286	0.030*

All data are presented with 95% confidence intervals. * p < 0.05, ** p < 0.01, *** p < 0.001

Abbreviations: EQ-5D-5L, five-level EuroQol 5-dimension; K-WOMAC, Korean Western Ontario and McMaster; MCS, mental composite summary; NRS, numeric rating score; PCS, physical composite summary; PPT, pharmacopuncture; PT, physical therapy; VAS, visual analog scale

Table S3. Primary and secondary outcomes after treatment at each timepoint (MI ANCOVA)

		Week 1-1	Week 1-2	Week 2-1	Week 2-2	Week 3-1	Week 3-2	Week 4	Week 6
NRS score	PPT group	5.87 (5.82–5.93)	5.41 (5.04–5.77)	5.17 (4.73–5.61)	4.69 (4.22–5.15)	3.87 (3.35–4.39)	3.37 (2.82–3.91)	3.17 (2.57–3.78)	3.16 (2.49–3.83)
	PT group		5.96 (5.61–6.32)	5.46 (5.05–5.88)	5.55 (5.10–6.00)	5.07 (4.56–5.58)	5.05 (4.52–5.58)	5.18 (4.60–5.77)	5.20 (4.55–5.85)
	Difference	—	–0.56 (–1.07 to –0.04)	–0.30 (–0.91 to 0.31)	–0.86 (–1.51 to –0.22)	–1.20 (–1.93 to –0.47)	–1.68 (–2.45 to –0.92)	–2.01 (–2.86 to –1.16)	–2.04 (–2.98 to –1.10)
	p-value	—	0.034*	0.330	0.010*	0.002**	< 0.001***	< 0.001***	< 0.001***
VAS score	PPT group	59.18 (58.65–59.71)		51.56 (47.35–55.77)		38.69 (33.48–43.91)		31.04 (25.00–37.07)	32.85 (24.68–41.02)
	PT group			55.26 (51.29–59.23)		50.89 (45.81–55.97)		52.02 (46.14–57.89)	51.65 (44.29–59.01)
	Difference	—		–3.70 (–9.54 to 2.15)		–12.20 (–19.54 to –4.86)		–20.98 (–29.46 to –12.50)	–18.80 (–30.07 to –7.52)
	p-value	—		0.208		0.002**		< 0.001***	0.002**
K-WOMAC score	PPT group	59.31 (58.07–60.55)						42.98 (36.98–48.98)	41.52 (35.29–47.75)
	PT group							56.22 (50.37–62.06)	54.90 (48.83–60.97)

	Difference	—		–13.23 (–21.66 to –4.81)	–13.38 (–22.13 to –4.63)
	p-value	—		0.003**	0.004**
PCS score	PPT group	39.62 (39.19–40.05)		43.46 (41.00–45.92)	44.41 (41.45–47.38)
	PT group			42.08 (39.69–44.48)	41.94 (39.06–44.83)
	Difference	—		1.37 (–2.08 to 4.82)	2.47 (–1.68 to 6.62)
	p-value	—		0.425	0.235
MCS score	PPT group	51.48 (50.85–52.12)		53.27 (50.37–56.17)	54.85 (51.63–58.08)
	PT group			51.89 (49.07–54.71)	51.00 (47.86–54.14)
	Difference	—		1.38 (–2.74 to 5.50)	3.85 (–0.74 to 8.44)
	p-value	—		0.502	0.097
EQ-5D-5L score	PPT group	0.73 (0.72–0.73)		0.81 (0.78–0.84)	0.81 (0.77–0.86)
	PT group			0.78 (0.75–0.81)	0.75 (0.71–0.80)

Difference	—		0.03 (–0.02 to 0.07)	0.06 (0.00–0.12)
p-value	—		0.196	0.064

All data are presented with 95% confidence intervals. * $p < 0.05$, ** $p < 0.01$, *** $p < 0.001$

Abbreviations: EQ-5D-5L, five-level EuroQol 5-dimension; K-WOMAC, Korean Western Ontario and McMaster; MCS, mental composite summary; MI ANCOVA, multiple imputation analysis of covariance; NRS, numeric rating score; PCS, physical composite summary; PPT, pharmacopuncture; PT, physical therapy; VAS, visual analog scale

Table S4. Primary and secondary outcomes after treatment at each timepoint (LOCF ANCOVA)

		Week 1-2	Week 2-1	Week 2-2	Week 3-1	Week 3-2	Follow-up<br>Week 4	Follow-up<br>Week 6
NRS score	PPT group	5.41 (5.04–5.77)	5.13 (4.69–5.57)	4.69 (4.22–5.15)	3.87 (3.35–4.39)	3.37 (2.82–3.91)	3.17 (2.57–3.78)	3.16 (2.49–3.83)
	PT group	5.96 (5.61–6.32)	5.48 (5.07–5.90)	5.55 (5.10–6.00)	5.07 (4.56–5.58)	5.05 (4.52–5.58)	5.18 (4.60–5.77)	5.20 (4.55–5.85)
	Difference	–0.56 (–1.07 to –0.04)	–0.35 (–0.96 to 0.26)	–0.86 (–1.51 to –0.22)	–1.20 (–1.93 to –0.47)	–1.68 (–2.45 to –0.92)	–2.01 (–2.86 to –1.16)	–2.04 (–2.98 to –1.10)
	p-value	0.034*	0.251	0.010*	0.002**	< 0.001***	< 0.001***	< 0.001***
VAS score	PPT group		51.13 (46.99–55.26)		38.69 (33.48–43.91)		31.04 (25.00–37.07)	31.81 (22.63–40.98)
	PT group		55.49 (51.57–59.41)		50.89 (45.81–55.97)		52.02 (46.14–57.89)	50.45 (41.59–59.31)
	Difference		–4.36 (–10.13 to 1.40)		–12.20 (–19.54 to –4.86)		–20.98 (–29.46 to –12.50)	–18.64 (–31.44 to –5.85)
	p-value		0.133		0.002**		< 0.001***	0.006**
K-WOMAC score	PPT group						42.98 (36.98–48.98)	41.52 (35.29–47.75)
	PT group						56.22 (50.37–62.06)	54.90 (48.83–60.97)

	Difference		–13.23 (–21.66 to -4.81)	–13.38 (–22.13 to –4.63)
	p-value		0.003**	0.004**
PCS score	PPT group		43.46 (41.00–45.92)	44.41 (41.45–47.38)
	PT group		42.08 (39.69–44.48)	41.94 (39.06–44.83)
	Difference		1.37 (–2.08 to 4.82)	2.47 (–1.68 to 6.62)
	p-value		0.425	0.235
MCS score	PPT group		53.27 (50.37–56.17)	54.85 (51.63–58.08)
	PT group		51.89 (49.07–54.71)	51.00 (47.86–54.14)
	Difference		1.38 (–2.74 to 5.50)	3.85 (–0.74 to 8.44)
	p-value		0.502	0.097
EQ-5D-5L score	PPT group		0.81 (0.78–0.84)	0.81 (0.77–0.86)
	PT group		0.78 (0.75–0.81)	0.75 (0.71–0.80)
	Difference		0.03 (–0.02 to 0.07)	0.06 (0.00–0.12)

p-value		0.196	0.064

Note: All data are presented with 95% confidence intervals. * $p < 0.05$, ** $p < 0.01$, *** $p < 0.001$

Abbreviations: ANCOVA, analysis of covariance; EQ-5D-5L, five-level EuroQol 5-dimension; K-WOMAC, Korean Western Ontario and McMaster; LOCF, last observation carried forward; MCS, mental composite summary; NRS, numeric rating score; PCS, physical composite summary; PPT, pharmacopuncture; PT, physical therapy; VAS, visual analog scale;

Table S5. Laboratory test results before and after the intervention

	PPT group			PT group		
	Before	After	p-value	Before	After	p-value
**WBC count**	5.91 ± 1.29	5.96 ± 1.27	0.838	6.12 ± 1.93	5.79 ± 1.51	0.470
**NEUT count**	52.71 ± 5.21	52.98 ± 7.55	0.828	55.32 ± 9.53	55.31 ± 10.63	0.995
**LYMPO count**	36.44 ± 6.00	36.65 ± 8.05	0.850	35.14 ± 9.35	34.71 ± 10.46	0.799
**MONO count**	6.04 ± 1.50	5.61 ± 1.64	0.053	5.24 ± 1.40	5.20 ± 1.49	0.901
**Eosinophil count**	4.05 ± 1.88	4.09 ± 1.32	0.913	3.68 ± 1.97	4.08 ± 2.38	0.152
**Basophil count**	0.76 ± 0.28	0.67 ± 0.18	0.187	0.62 ± 0.27	0.71 ± 0.41	0.157
**RBC count**	4.68 ± 0.38	4.64 ± 0.43	0.402	4.55 ± 0.31	4.56 ± 0.36	0.794
**Hgb level**	14.17 ± 1.34	14.27 ± 1.58	0.424	13.84 ± 0.87	14.03 ± 1.00	0.244
**Hct level**	42.95 ± 3.67	42.57 ± 4.25	0.372	41.96 ± 2.35	42.09 ± 3.01	0.744
**MCV level**	91.82 ± 4.36	91.68 ± 4.25	0.444	92.28 ± 2.74	92.29 ± 2.77	0.933
**MCH level**	30.29 ± 1.85	30.71 ± 1.94	0.046	30.42 ± 1.07	30.74 ± 1.27	0.284
**MCHC level**	32.97 ± 0.99	33.49 ± 1.28	0.026	32.96 ± 0.63	33.31 ± 1.16	0.249
**Platelet count**	226.17 ± 53.85	236.22 ± 58.20	0.018	235.42 ± 48.68	230.68 ± 42.05	0.573
**ESR**	13.33 ± 10.74	14.56 ± 12.95	0.563	15.63 ± 10.54	16.11 ± 10.05	0.713
**T-Protein level**	7.27 ± 0.38	7.08 ± 0.38	0.026	7.15 ± 0.33	7.12 ± 0.31	0.620
**Albumin level**	4.49 ± 0.22	4.49 ± 0.27	0.948	4.44 ± 0.20	4.45 ± 0.22	0.678
**T-Bilirubin level**	0.70 ± 0.28	0.71 ± 0.22	0.913	0.58 ± 0.21	0.68 ± 0.31	0.104
**ALP level**	198.07 ± 60.37	190.12 ± 51.81	0.219	190.16 ± 67.24	187.19 ± 54.81	0.674
**AST level**	28.75 ± 18.60	23.29 ± 6.21	0.184	18.96 ± 5.00	18.77 ± 4.14	0.780
**ALT level**	23.17 ± 11.10	23.09 ± 12.83	0.961	19.34 ± 8.07	17.01 ± 6.52	0.041
**r-GTP level**	27.56 ± 18.73	29.72 ± 25.03	0.299	21.74 ± 12.43	20.68 ± 12.35	0.377
**BUN level**	14.48 ± 5.01	14.00 ± 4.02	0.560	14.03 ± 5.91	13.64 ± 4.73	0.574
**Cr level**	0.76 ± 0.14	0.75 ± 0.15	0.815	0.70 ± 0.15	0.69 ± 0.15	0.430
**CRP level**	0.11 ± 0.20	0.05 ± 0.09	0.165	0.08 ± 0.09	0.24 ± 0.73	0.334

Abbreviations: ALP, alkaline phosphatase; ALT, alanine aminotransferase; AST, aspartate Aminotransferase; BUN, blood urea nitrogen; Cr, creatinine; ESR, erythrocyte sedimentation rate; Hct, hematocrit; Hgb, hemoglobin; LYMPO, lymphocyte; MCH, mean corpuscular hemoglobin; MCHC, mean corpuscular hemoglobin concentration; MONO, monocyte; MCV, mean cell volume; NEUT, neutrophil; PPT, pharmacopuncture; PT, physical therapy; RBC, red blood cell; r-GTP, r-glutamyl transferase; WBC, white blood cell

Table S6. Ratios of abnormality in the laboratory test results

	PPT group			PT group		
	Before	After	p-value	Before	After	p-value
**WBC count**	1 (5.6)	-	-	2 (10.5)	2 (10.5)	1.000
**NEUT count**	5 (27.8)	8 (44.4)	0.453	6 (31.6)	9 (47.4)	0.453
**LYMPO count**	4 (22.2)	6 (33.3)	0.625	6 (31.6)	9 (47.4)	0.250
**MONO count**	2 (11.1)	2 (11.1)	1.000	1 (5.3)	1 (5.3)	1.000
**Eosinophil count**	4 (22.2)	4 (22.2)	1.000	3 (15.8)	3 (15.8)	1.000
**Basophil count**	2 (11.1)	1 (5.6)	1.000	1 (5.3)	1 (5.3)	1.000
**RBC count**	1 (5.6)	1 (5.6)	1.000	-	-	-
**Hgb level**	2 (11.1)	3 (16.7)	1.000	-	1 (5.3)	-
**Hct level**	1 (5.6)	1 (5.6)	1.000	-	-	-
**MCV level**	-	-	-	-	-	-
**MCH level**	1 (5.6)	1 (5.6)	1.000	-	-	-
**MCHC level**	1 (5.6)	1 (5.6)	1.000	-	1 (5.3)	-
**Platelet count**	-	-	-	-	-	-
**ESR**	7 (38.9)	4 (22.2)	0.250	6 (31.6)	7 (36.8)	1.000
**T-Protein level**	1 (5.6)	-	-	-	-	-
**Albumin**	-	-	-	-	-	-
**T-Bilirubin**	1 (5.6)	-	-	1 (5.3)	2 (10.5)	1.000
**ALP level**	-	-	-	3 (15.8)	1 (5.3)	0.500
**AST level**	2 (11.1)	-	-	-	-	-
**ALT level**	2 (11.1)	2 (11.1)	-	-	-	-
**r-GTP level**	1 (5.6)	1 (5.6)	1.000	-	-	-
**BUN level**	3 (16.7)	3 (16.7)	1.000	7 (36.8)	1 (5.3)	0.031
**Cr level**	1 (5.6)	1 (5.6)	1.000	-	-	-
**CRP level**	1 (5.6)	-	-	-	1 (5.3)	-

Abbreviations: ALP, alkaline phosphatase; ALT, alanine Aminotransferase; AST, aspartate aminotransferase; BUN, blood urea nitrogen; Cr, creatinine; Hct, hematocrit; Hgb, hemoglobin; LYMPO, lymphocyte; MCH, mean corpuscular

hemoglobin; MCHC, mean corpuscular hemoglobin concentration; MCV, mean cell volume; MONO, monocyte; NEUT, neutrophil; ESR, erythrocyte sedimentation rate; RBC, red blood cell; r-GTP, r-glutamyl transferase; PPT, pharmacopuncture; PT, physical therapy; WBC, white blood cell
